# A Case of Follicular Bronchiolitis as the Histological Counterpart to Nodular Opacities in Bronchiectatic *Mycobacterium avium* Complex Disease

**DOI:** 10.1155/2012/214601

**Published:** 2012-10-24

**Authors:** Kentaro Wakamatsu, Nobuhiko Nagata, Kazuhito Taguchi, Kouji Takakura, Chika Harada, Hiroyuki Kumazoe, Masayuki Kawasaki

**Affiliations:** ^1^Division of Respiratory Medicine, National Hospital Organization Omuta Hospital, 1044-1 Oaza Tachibana, Omuta City 837-0911, Japan; ^2^Department of Respiratory Medicine, Fukuoka University Chikushi Hospital, 1-1-1 zokumyouin, Chikucino City 818-8502, Japan; ^3^Division of Radiology, National Hospital Organization Omuta Hospital, 1044-1 Oaza Tachibana, Omuta City 837-0911, Japan

## Abstract

Here we report the case of a 72-year-old woman with nodular bronchiectatic *Mycobacterium avium* complex (MAC) disease. Chest computed tomography on admission revealed multiple micronodular and branching opacities in both lobes with segmental distribution; bronchiectasis and bronchial wall thickening were observed in the middle lobe and lingula. The patient consented to and underwent thoracoscopic lung biopsy; epithelioid granulomas were occasionally observed, but follicular bronchiolitis was widespread. While bronchial lesions from nontuberculous mycobacterial infection generally present as epitheliod granulomas, the present case suggests that follicular bronchiolitis can also be a histological counterpart to nodular opacities in nodular bronchiectatic MAC disease.

## 1. Introduction

The estimated prevalence of pulmonary nontuberculous mycobacterial (NTM) infection in Japan is more than 5.7 per 100,000 population, a high level even by international standards [1]. Of particular interest is the recent increase in nodular bronchiectatic *Mycobacterium avium *complex (MAC) disease [1–3]. While previous reports have revealed these bronchial lesions to be histologically granulomatous after operation of nodular bronchiectatic MAC pharmacotherapy [4–7], there have been those which examine radiological-histological correlation on the nodules before treatment. Here we report a case in which follicular bronchiolitis was the histological counterpart to nodular opacities of nodular bronchiectatic MAC disease as assessed by diagnostic biopsy. 

## 2. Case Report

The patient was a 72-year-old woman with chief complaints of coughing and sputum. Her medical history revealed hypertension from 10 years before; family history was unremarkable. Social history was as follows: smoking (no), drinking (social), allergies (none), pet ownership history (none), use of down blankets (no), use of humidifier (yes), use of Kampo medicine or health foods (no), dust inhalation history (none), and occupation (seaweed harvesting). 

### 2.1. Current Medical History

Despite being informed of abnormal opacities on a chest X-ray in July 2004, she refused treatment. On April 10, 2006, she experienced a 38°C fever and headache (no coughing or sputum) and visited a local clinic the next day. Chest X-ray revealed abnormal opacities; white blood cell (WBC) 9800 and C-reactive protein (CRP) 6+ suggested a pronounced inflammatory response. She was diagnosed with pneumonia and administered levofloxacin (LVFX), clarithromycin (CAM), and ceftriaxone (CTRX). Inflammation resolved by April 15 (WBC 4800 and CRP 1+), although abnormal opacities remained on the chest X-ray. Right chest pain, coughing, and sputum (no fever) appeared on May 25. She was administered CTRX upon revisiting the clinic, but WBC (10500) and CRP (6+) did not improve. She was referred to our hospital and admitted on May 29.

The patient was alert on admission. Characteristics were as follows: height 142.0 cm, weight 41.0 kg. BMI 20.9 kg/m^2^, blood pressure 144/93 mmHg, pulse 82 beats per min, and body temperature 35.7°C. There was no palpable swelling in the superficial lymph nodes, and no signs of skin lesions, edema, cyanosis, or head or neck abnormalities. Heart and breathing sounds were normal. Liver and spleen were not palpable. There were no neurological findings.

**Figure 1:**
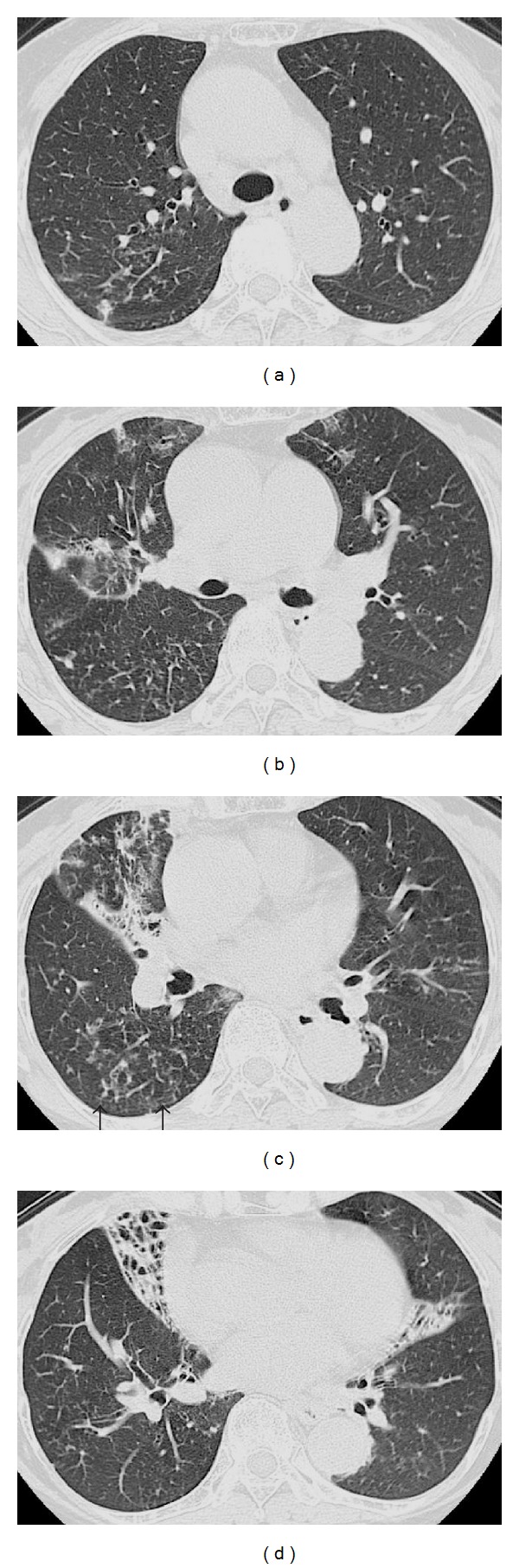
High-resolution CT revealed centrilobular micronodular opacities (right S^2^ and S^3^, right S^6^ and S^9^, and left upper lobe) (a, b, and c) and volume loss with bronchial wall thickening and bronchiectatic changes in both the right middle lobe and left lingual (d). Biopsied specimens were obtained from the area indicated by arrows (c).

Findings on admission are summarized in Table 1. WBC and biochemical tests were normal, but CRP was high (2.1 mg/dL). Rheumatoid factor and antinuclear antibodies were mildly increased. Chest X-ray on admission revealed nodular and infiltrative opacities in both lung fields. Chest CT on admission revealed multiple micronodular and branching opacities presenting with segmental distribution in right S^2^ and S^3^, right S^6^ and S^9^, and the left upper lobe (Figure 1(a)). There was increased lung density with volume loss in the middle lobe and lingula, as well as bronchiectasis and bronchial wall thickening. 

### 2.2. Progress after Admission

**Figure 2:**
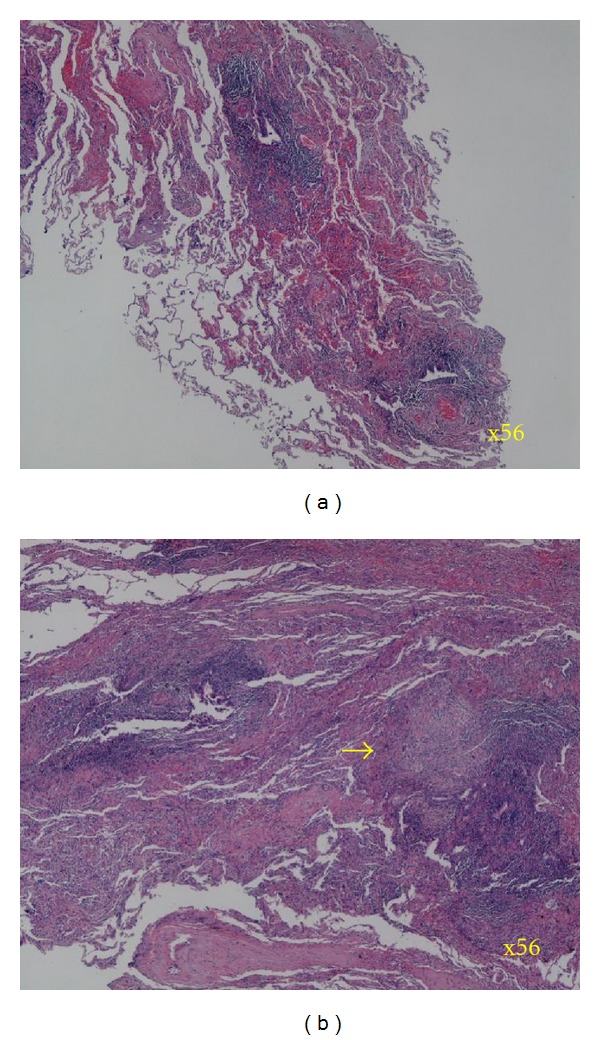
Lung biopsy stained with hematoxylin-eosin showed many areas of follicular bronchiolitis characterized by the presence of hyperplastic lymphoid follicles distributed along the bronchioles and few granulomas (arrow) (original magnification ×56).

NTM infection was suspected from imaging findings on admission. Sputum cultures were negative for three consecutive days. Bronchofiberscopy on June 7 revealed no abnormal findings in the visible range. Sputum smears remained negative for acid fast bacilli (AFB) after bronchial washing of right B^2^, B^5^, and B^6^ and bronchial brushing of B^2^ and B^5^. After conferring with the patient, thoracoscopic lung biopsy of S^6^ was carried out on June 22 for definitive diagnosis (Figure 1(c)). While epithelioid granulomas with occasional Langhans giant cells were observed (Figure 2), the main finding was fibrosis and lymphocytic aggregates in the airways, that is, follicular bronchiolitis. Lung specimens were AFB smear-negative. On June 7, bronchial lavage fluid cultures were AFB smear positive; *M. intracellulare* was confirmed by polymerase chain reaction (PCR). The patient was placed on watchful waiting; chest X-ray and CT on June 21 revealed strengthening of micronodular densities. She underwent bronchoscopy on August 21, 2007. AFB smears were positive after bronchial washing of right B^2^, B^5^, and B^6^ and bronchial scraping of right B^2^ and B^5^. PCR on bronchial lavage fluid confirmed the presence of *M. intracellulare. *On August 21, 2007, she was started on rifampicin 450 mg/day, ethambutol (EB) 500 mg/day, CAM 600 mg/day, and amikacin (AMK) 400 mg/day. Abnormal findings on CT and X-rays (e.g., bronchial lesions) subsequently improved.

## 3. Discussion

Among the various types of pulmonary MAC disease are nodular bronchiectatic type, fibrocavitary type, hot tub lung type, and disseminated type [8, 9]. The recent rise of pulmonary MAC disease in Japan can be attributed to the nodular bronchiectatic type [1, 2]. This type had been reported by Yamamoto in 1970 as bronchiectatic and by Shimoide in 1980 as the middle lobe/lingual type or the chronic bronchitis type [10, 11]. Yet, it was only after Prince et al. reported its clinical manifestations in 1989 that the disease received widespread attention [3]. According to Prince et al., the nodular bronchiectatic type occurs frequently in middle-aged women and is associated with persistent cough, purulent sputum, predominant slowly progressive nodular opacities, and, albeit rarely, cavitary disease. 

MAC classically involves the middle lobe and lingula, and in many cases, it can be bilateral from the outset. Imaging typically reveals the presence of micronodules and its aggregation and consolidation in peripheral (subpleural) lung fields, drainage bronchus wall thickening and bronchiectasis, and lung volume loss [12]. Lesions have a strong tendency for S^5^ than S^4^, and the cavity formation is rarely observed in the same lobe [4]. When involving surrounding lobes, lesions commonly spread to S^2^, S^3^, and S^6^. Although cavities are formed with bronchogenic spread of lesions as in tuberculosis, the cavity rarely enlarges. Lymph node and pleural lesions are also rare [13]. In the present case, chest CT revealed multiple micronodular and branching opacities presenting with segmental distribution in right S^2^ and S^3^, right S^6^ and S^9^, and the left upper lobe. There was also increased lung density associated with volume loss in the middle lobe and lingula, as well as bronchiectasis and bronchial wall thickening. These findings suggest that the present case is typical of nodular bronchiectatic type.

Fujita et al. reported the analysis of resected lung specimens from 5 patients with drug-administered fibrocavitary MAC disease, finding that nodules <5 mm on CT were 2-3 mm granulomas consisting of mononuclear and epithelioid cells [14]. Inflammatory infiltration of alveolar walls and caseous necrosis in the center of the granulomas in nodules >10 mm, lymphocyte infiltration in the bronchial submucosa extending from the bronchioli to acini, and extensive epithelioid cell infiltration have also been reported [5, 14]. Hebisawa et al. reported macroscopic and pathological findings from surgical specimens obtained from drug-administered patients with nodular bronchiectatic MAC disease and concluded that the most characteristic lesion is bronchiectasis. Epithelioid cell granulomas are occasionally observed within the high degree of small round cell infiltration, and airway ulcers are also seen in the thickened airway wall. Hebisawa et al. further reported granulomatous bronchitis and bronchiolitis associated with obstruction and stenosis of the bronchial lumen due to proliferative and sclerosing granuloma at the dilated distal bronchi, and a few nonspecific bronchiolitis associated with follicular bronchiolitis and small round cell infiltration with prominent lymph follicles [4, 6, 7]. In the present case, granulomatous lesions were minimal, and the main bronchiolar lesion was follicular bronchiolitis. The discrepancy with previous reports can be explained by the fact that Fujita et al. analyzed nodular lesions in fibrocavitary MAC disease, and Hebisawa et al. analyzed nodular opacities associated with severe bronchiectatic change. Thus, these studies may not reflect the histopathological features of centrilobular nodular opacities often seen in nodular bronchiectatic MAC disease by CT. In addition, the pathological specimens were obtained before treatment in our case, while those studies used resected specimens from drug-administered patients.

Many potential causes of follicular bronchiolitis have been reported, including connective tissue diseases such as rheumatoid arthritis and Sjogren's syndrome, immunodeficiency, hypersensitivity disorders such as hypereosinophilic syndrome, and chronic airway inflammation [15, 16]. There were no findings suggestive of connective tissue disorder or immunodeficiency in the present case, and given the lack of increased eosinophils, this strongly suggested the possibility of follicular bronchiolitis due to chronic airway inflammation by* M. intracellulare*. 

To our knowledge, this is a first report on the pathological examination of biopsies of centrilobular nodular lesions in nodular bronchiectatic MAC disease prior to therapy. 

## Figures and Tables

**Table 1:** Laboratory findings.

Hematology	
WBC	4500/*μ*L
Neut	63.5%
Lymp	24.6%
Mono	9.7%
Eos	1.8%
Baso	0.4%
RBC	457 × 10^4^/*μ*L
Hb	14.2 g/dL
Ht	44.1%
Plt	16.5 × 10^4^/*μ*L

Serology	

CRP	2.10 mg/dL
RAPA	×80
ANA	×80
Anti HTLV-1	Negative

Biochemistry	

TP	7.2 g/dL
Alb	4.3 g/dL
T-bil	0.5 mg/dL
ZTT	17.1 K-U
ChE	260 U/L
AST	20 IU/L
ALT	15 IU/L
ALP	270 U/L
*γ*-GTP	18 IU/L
LDH	154 IU/L
Glu	130 mg/dL
T-chol	153 mg/dL
TG	74 mg/dL
UA	3.9 mg/dL
BUN	15 mg/dL
Cr	0.44 mg/dL
Na	138 mEq/L
K	4.2 mEq/L
Cl	102 mEq/L

Arterial blood gas analysis	

pH	7.385
PaCO_2_	50.3 torr
PaO_2_	83.5 torr
SaO_2_	94.3%

Lung function test	

VC	1.82 L (88.3%)
FVC	1.74 L (84.5%)
FEV_1.0_	1.40 L (106.9%)
FEV_1.0%_	80.5%
